# Improved photoresponse with enhanced photoelectric contribution in fully suspended graphene photodetectors

**DOI:** 10.1038/srep02791

**Published:** 2013-09-27

**Authors:** Vikram Patil, Aaron Capone, Stefan Strauf, Eui-Hyeok Yang

**Affiliations:** 1 Department of Mechanical Engineering, Stevens Institute of Technology Castle Point on the Hudson, Hoboken, NJ 07030, USA; 2 Department of Physics & Engineering Physics, Stevens Institute of Technology Castle Point on the Hudson, Hoboken, NJ 07030, USA

## Abstract

Graphene's unique optoelectronic properties are promising to realize photodetectors with ultrafast photoresponse over a wide spectral range from far-infrared to ultraviolet radiation. The underlying mechanism of the photoresponse has been a particular focus of recent work and was found to be either photoelectric or photo-thermoelectric in nature and enhanced by hot carrier effects. Graphene supported by a substrate was found to be dominated by the photo-thermoelectric effect, which is known to be an order of magnitude slower than the photoelectric effect. Here we demonstrate fully-suspended chemical vapor deposition grown graphene microribbon arrays that are dominated by the faster photoelectric effect. Substrate removal was found to enhance the photoresponse by four-fold compared to substrate-supported microribbons. Furthermore, we show that the light-current input/output curves give valuable information about the underlying photophysical process responsible for the generated photocurrent. These findings are promising towards wafer-scale fabrication of graphene photodetectors approaching THz cut-off frequencies.

Graphene's high carrier mobility combined with its universal optical absorption from terahertz to the ultraviolet wavelength regime, as well as its large nonlinear optical coefficients, are promising towards novel applications in photodetection^1^,^2^,^3^,^4^,^5^, nonlinear optics^6^, plasmonics^7^,^8^, and broadband optoelectronics^8^,^9^,^10^,^11^,^12^. Efficient design of graphene-based optoelectronic devices requires an understanding of the main mechanisms contributing to the observed photocurrent. The light-matter interaction in exfoliated graphene was studied extensively and found to be either of photoelectric or photo-thermoelectric origin, depending on the sample geometry, electrode materials, or biasing^1^,^2^,^3^,^4^,^5^,^12^,^13^,^14^,^15^,^16^,^17^,^18^,^19^,^20^,^21^,^22^. In particular, the photoresponse in samples with closely spaced metal electrodes was attributed to the photoelectric effect driven by the band bending at the contacts^1^,^2^,^3^,^9^,^12^, while junctions between monolayer and bilayer graphene flakes^13^ as well as p-n junctions created via electrostatic gating are found to be dominated by the photo-thermoelectric effect^15^,^16^,^17^,^18^,^19^. The generated photocurrent is further enhanced by contributions from hot carriers that are generated by the optical excitation into energy states far above the Fermi level. These hot carriers are important since their relaxation via acoustic phonons displays a bottleneck effect^18^,^19^,^20^,^21^, and since their relaxation via electron-electron scattering creates carrier multiplication^22^,^23^,^24^,^25^, leading to a strong decoupling between electron and lattice temperature.

Practical applications require scalable approaches such as chemical vapor deposition (CVD) grown graphene photodetectors. The photophysics of CVD-grown graphene devices is however less understood, and it is a priori unclear to what extent inherent grain boundaries and other defects affect the mechanism of photocurrent generation. A recent time-resolved study of the photoresponse in CVD-grown graphene embedded in a coplanar stripeline circuit reveals that the time constant of the photo-thermoelectric effect is, at 130 ps, more than an order of magnitude slower than the photoelectric effect, at 4 ps^26^. It would, thus, be beneficial to design graphene photodetectors in which the photoelectric effect dominates the photoresponse. In addition to optimizing the response time, one also needs to maximize the magnitude of the photoresponsitivity, which is limited in substrate-supported graphene to a few mA/W^1^,^2^,^9^,^12^,^15^,^16^,^20^,^21^. Improvements in photoresponsitivity have been demonstrated using techniques such as hybrid graphene structures^27^ and quantum dots^28^. Very recently, exfoliated graphene that was partially suspended in air was found to improve the photoresponsitivity ten-fold, but the slower photo-thermoelectric effect still dominates the photoresponse in such a device configuration^29^.

Here we demonstrate fully-suspended CVD-grown graphene microribbon arrays that are dominated by the faster photoelectric effect. Removal of the substrate was found to enhance the photoresponse by four-fold compared to substrate-supported microribbons. We also show that the slope of the light-current input/output curves (L-I curves) gives valuable information about the underlying photophysical process responsible for time-integrated photocurrents. These findings are promising towards wafer-scale fabrication of graphene photodetectors approaching THz cut-off frequencies.

## Results

### Device fabrication, characterization and measurements

**Figure 1:**
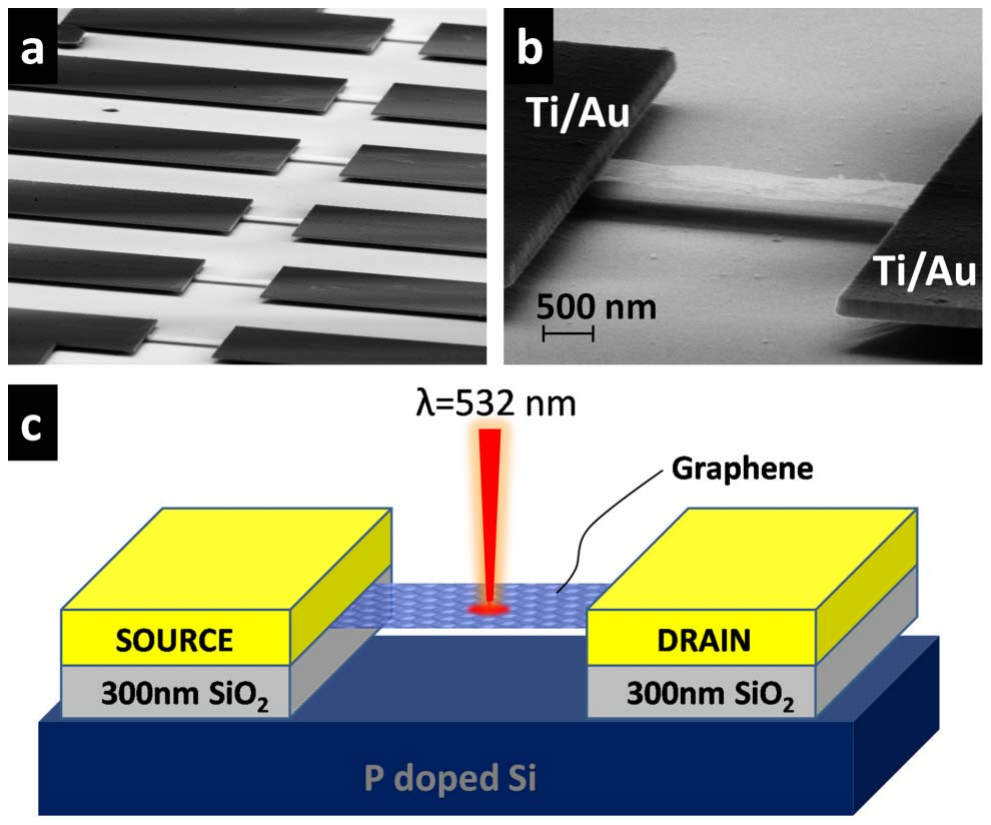
CVD-grown graphene photodetector devices. (a) SEM images showing an array of fully suspended graphene photodetectors fabricated on large area CVD graphene on a 300 nm layer of SiO_2_thermally grown on p-type silicon substrate. (b) SEM image of individual photodetector device with well suspended graphene microribbon contacted with Ti/Au metal contacts on both sides. (c) Schematic of the suspended graphene device during the photocurrent measurements.

In order to compare the photoresponse of fully suspended graphene microribbons with substrate-supported ones, we fabricated devices using large area CVD-grown graphene which was transferred onto p-type silicon wafers with 300 nm thick thermally grown SiO_2_ and patterned using electron-beam lithography (For details, see Method Section). The graphene quality was evaluated by Raman spectroscopy as well as optical transmission measurements and found to be monolayer graphene (see Supplementary Fig. S1, S2). For suspending graphene, the fabricated devices were then undercut with buffered oxide etchant (BOE) solution resulting in 5 μm long graphene microribbons suspended between metal contacts as shown in Fig. 1a,b. The photoresponse was measured on the same device before and after it was suspended in order to avoid effects from spatial inhomogeneity in the CVD material. Figure 1c shows the schematic of the device configuration for photocurrent measurements, which were carried out using a standard lock-in technique and by spatial scanning of a 532 nm laser diode operating in continuous mode (For details see Methods Section).

**Figure 2:**
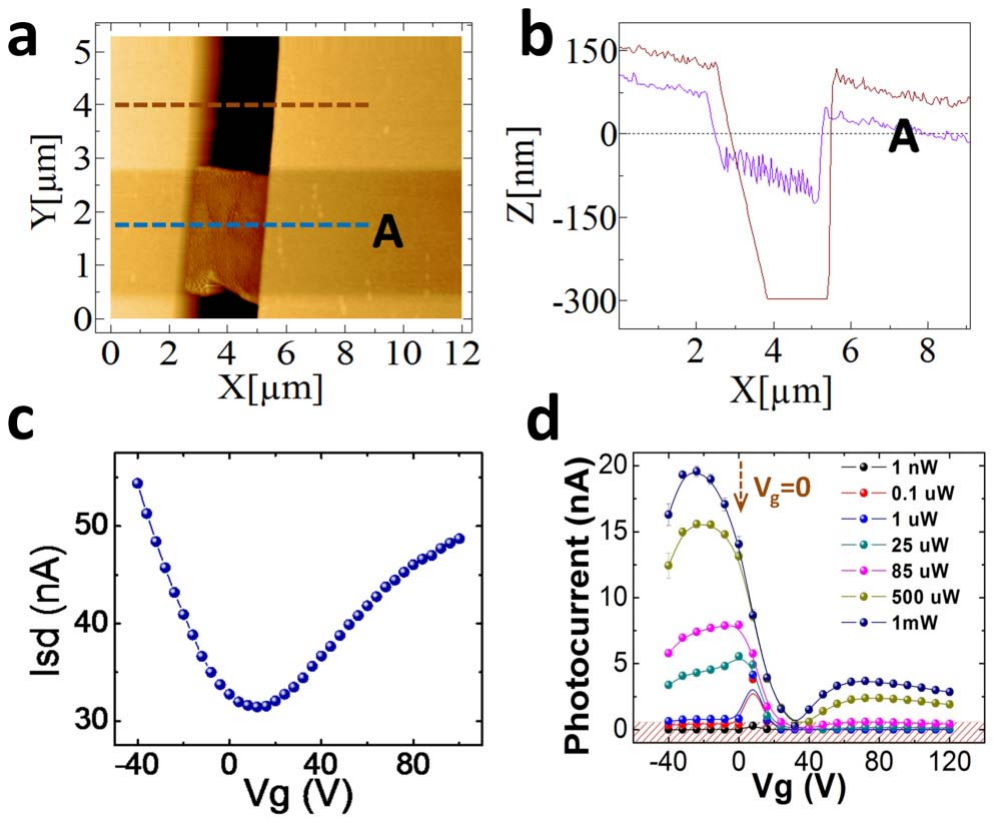
Structural, electronic, and optoelectronic characterization of the graphene photodetector devices. (a) AFM scans over a suspended graphene ribbon. (b) Corresponding height map showing that the suspended graphene area is about 250 nm above the silicon substrate. (c) Backgate sweep without light at constant source drain bias of 5 mV showing Dirac point near +15 V indicating slight p-type graphene. Data recorded after current annealing for several hours and stable response. (d) Photocurrent measured on the fully supported graphene microribbon with varying backgate at different incident powers. The hashed region illustrates the instrument noise floor of about 50 pA.

The SEM image in Fig. 1b shows a shadow underneath the graphene microribbon which indicates that it is fully suspended. To quantify this better we recorded two AFM scans of another fully suspended graphene microribbon, one over graphene and the other over the area next to it as indicated by the dashed lines in Fig. 2a. The gap between the suspended graphene microribbon and the silicon surface was determined from Fig. 2b to be at least 250 nm, while the height of the metallization is 150 nm as expected. Both, SEM imaging and AFM scans confirm good suspension of the graphene microribbon without any obvious ripples or twisting that could cause current leakage to the substrate. As fabricated, the graphene microribbon on the SiO_2_ substrate exhibits heavy p-type doping with the Dirac point sitting beyond +100 V. It is observed that subsequent backgate sweeps are strongly affected by current-annealing effects giving rise to shifts of the Dirac point in each new scan. In order to strongly reduce such effects, we first current-annealed samples for several hours and confirmed that subsequent backgate sweeps are on top of each other. The resulting backgate sweep is shown in Fig. 2c and displays a Dirac point near +15 V, indicating that the unintentional p-type doping from device processing is strongly reduced, similar to previous findings^15^.

### Backgated photoresponse of supported graphene

In order to determine the device operation parameters giving rise to a maximal photocurrent response we have recorded the photocurrent as a function of incident optical pump power and back gate voltage varied from negative gate voltage to positive gate voltage. The corresponding data in Fig. 2d show that the highest photocurrent value is achieved under slight negative bias in the p-type region. Beyond the Dirac point, the values sharply drop by two orders of magnitude and display a minimum around 35 V, and recover partly beyond 60 V in the n-type regime. This behavior reflects the effect of electrical doping and the interplay of the Fermi level pinning at the Ti/graphene contacts, the applied moderate source-drain bias of V_SD_ = 100 mV, and the voltage tunable Fermi level of graphene. Since the work function of Ti is approximately 0.3 eV lower than that of graphene^30^, the source contact is strongly n-type, while the drain contact is moderately n-type with respect to non-gated graphene. At negative back gate voltages, the Fermi level of graphene is in the valence band and thus the effective lateral electric field required for carrier transport in the resulting n-p-n structure is maximized. The general V-shape of the photocurrent backgate sweep under optical illumination shown in Fig. 2d is similar to the V-shape backgate sweep of the source-drain current without illumination shown in Fig. 2c, and predominantly reflects the voltage tunable Fermi level of graphene, which follows the Dirac band structure. One apparent difference, however, is the magnitude of the photocurrent in the n-branch at positive back gate voltages, which is one order of magnitude smaller compared to the p-branch. This is in contrast to the values of source-drain currents in the n- and p-region in Fig. 2c, where the n-branch is only about two-fold smaller due to the lower mobility in the n-branch. The reason is that photocarriers are generated in pairs, i.e. an electron and a hole, upon absorption of a photon. Thus there is an additional loss channel due to the possibility of fast carrier recombination in this case, while the unipolar n-type carrier injection from the metal contact in biased graphene without illumination (Fig. 2c) is less affected by this loss channel. As a result, the significantly lower mobility in the n-branch and the particular n-p-n type band lineup from the Fermi level pinning at the Ti/graphene contacts in the graphene ribbons favors backgate biasing in the p-branch for highest photocurrent generation at fixed pump power.

To avoid any possible spatial actuation if the suspended graphene membrane is biased with a back gate^31^, we have chosen to carry out the comparison between supported and suspended devices at V_g_ = 0, since the device is close to optimum performance near this voltage setting as marked with an arrow in Fig. 2d

### Photoresponse of fully suspended graphene photodetector

**Figure 3:**
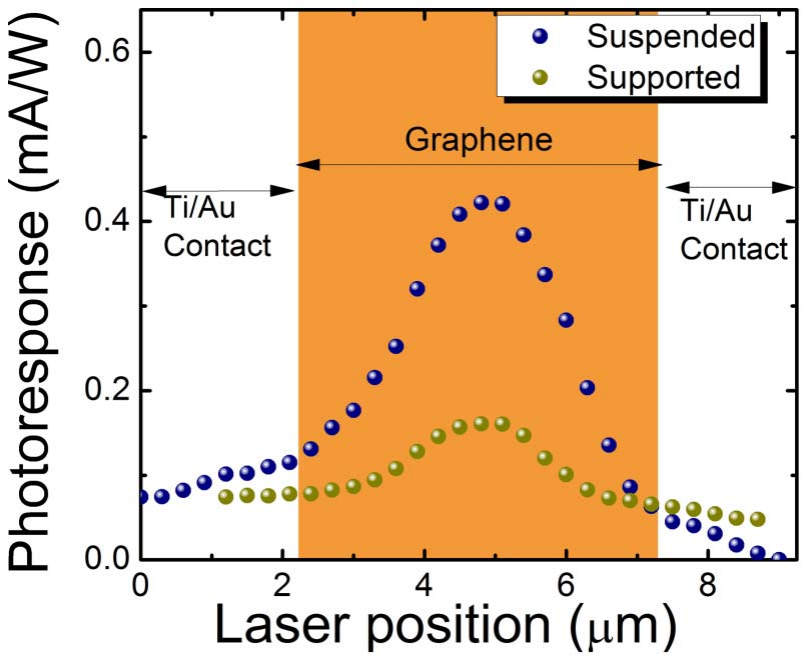
Spatial scans of photoresponse for substrate-supported (green dots) and suspended (blue dots) cases. The scan direction is along the length of the microribbon from contact to contact as indicated by the dashed line labeled “A” in Figure 2a. At the center position near 5 μm, a four-fold enhanced photoresponsitivity is found for the suspended graphene.

We now discuss the comparison of device performance of the fully suspended graphene microribbon with the supported one. To avoid any influence of varying quality of the CVD graphene material or residual doping, we compare the same device before and after etching the sacrificial SiO_2_ layer. The incident laser spot was scanned along the length of the graphene microribbon while recording the room-temperature photocurrent under V_SD_ = 100 mV, V_g_ = 0 V, from the sample held in vacuum. The scan direction between contacts is illustrated by the dashed line marked “A” in the AFM image of Fig. 2a. The photocurrent contribution ΔI_PC_ from the graphene layer was corrected by subtracting the background value from the total recorded photocurrent values. Figure 3 shows the photoresponse plotted against the laser position along the graphene microribbon for both cases. At the metal contacts, the photoresponse is sharply diminished compared to the photoresponse at the center of the 5 μm long microribbon. We attribute this dome-shape profile of the photocurrent to the particular Fermi-level bending at the Ti/graphene interface at both contacts. The behavior is similar to previous results with Ti/graphene interfaces at the contacts^12^, which is in contrast to asymmetric profiles with positive and negative current contributions observed with Pt-graphene interfaces^26^. Most importantly, when comparing the peak values in the center, we observed a four-fold improved photoresponse in the fully suspended case.

### Light-current input/output characteristic

**Figure 4:**
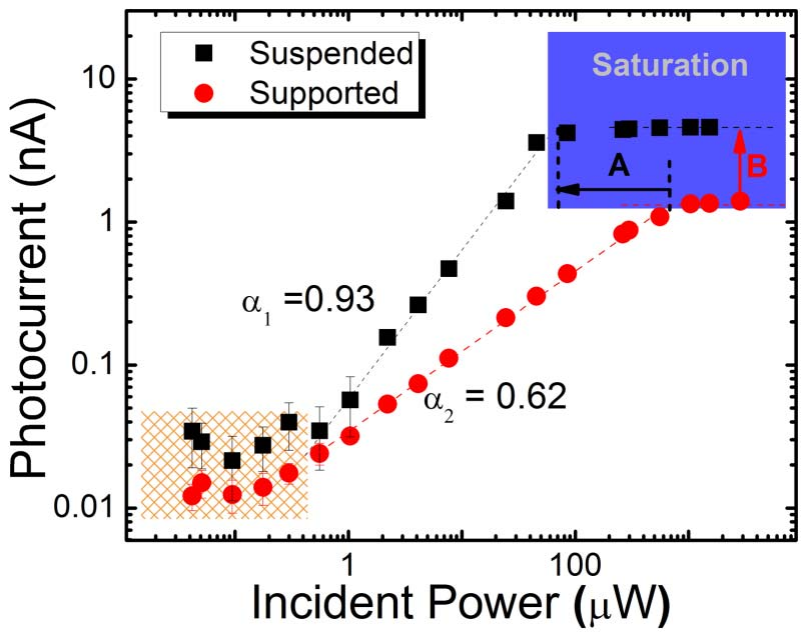
Light-current input/output curves. Photocurrent plotted against the incident laser power on a logarithmic scale showing enhanced photocurrent in the fully suspended graphene microribbons with significant slope change. The magnitude of the slope is indicative of the underlying photocurrent generation mechanism as detailed in the text. The black arrow (label A) indicates six-fold lower pump power levels for photocurrent saturation in the case of suspended graphene. The red arrow (label B) indicates four-fold increased photocurrents for suspended graphene in the saturation regime.

While demonstrating four-fold enhanced photocurrents in large-scale CVD-grown graphene photodetectors is of great technological importance, one would also like to understand the underlying photophysical mechanism to be able to optimize the detector device performance. The two main competing mechanisms for photocurrent generation in graphene are the photo-thermoelectric effect and the photoelectric effect. One way to tell these two effects apart is to characterize their time constant in optical pump-probe experiments which differ by at least an order of magnitude^26^. This technique requires, however, integration of our graphene microribbons into a coplanar stripeline circuit. In this study, we show that the slope of the L-I curve is an alternative way to characterize the dominant mechanism for photocurrent generation. To this end the photocurrent was measured on a fully suspended graphene device and a fully supported one, respectively, under variable power, continuous-wave optical excitation. Figure 4 shows the corresponding L-I curve on a logarithmic scale. Above the noise floor of approximately 50 pA, the photocurrent I_PC_ rises proportional with incident power (P_opt_). Ultimately, I_PC_ saturates at the highest pump power with values for suspended graphene being approximately 6-times smaller than those for supported graphene, as indicated by the arrow labeled “A” in Fig. 4. Above the noise floor and below saturation, the data follow a power law such that I_PC_ ∝ (P_opt_)^α^. For supported graphene we studied five different devices and found an average value of α = 0.62 ± 0.05 at zero gate voltage. In stark contrast, the power exponent of the fully suspended device is with 0.9, near unity. The pronounced change in slope of the L-I input-output curve indicates that the underlying physical mechanism has changed.

## Discussion

**Figure 5:**
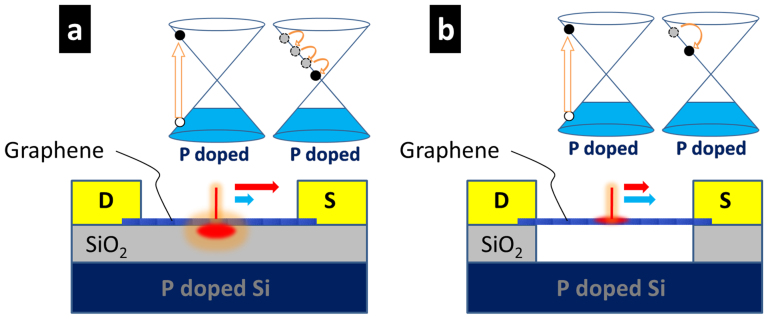
Schematic of the photocurrent generation process. (a) substrate-supported graphene devices and (b) fully suspended devices. Optical excitation at 2.3 eV creates hot carriers in the conduction band (left Dirac cones in a and b). Carriers either relax efficiently due to substrate scattering effects and low mobility (right Dirac cone in case a) or experience a slow-down in relaxation due to absence of substrate scattering and enhanced mobility/heat-conduction (right Dirac cone in case b). The length of the blue arrows schematically indicate the magnitude of the contribution from the photoelectric effect and the red arrows the magnitude of the photo-thermoelectric effect, for both cases respectively.

The power relationship between the photo-thermoelectric current (I_TE_) and the optical pump power P_opt_ can be estimated and yields I_TE_ ∝ (P_opt_)^2/3^, i.e. α = 0.66 (see supplementary information). Our experimental data for substrate-supported microribbons follow this trend closely with α = 0.62 ± 0.05, strongly indicating that the photo-thermoelectric contribution is the dominant effect, as was also reported by several groups^13^,^14^,^15^,^16^,^17^,^18^,^19^,^20^,^21^,^22^. Figure 5 illustrates the photocurrent generation process schematically. The optical interband absorption of the incident laser beam at 2.3 eV creates hot carriers with a temperature T_e_ underneath the laser excitation spot located in the center of the microribbon. These photoexcited carriers interact strongly with the surface polar phonons (SPP) of the substrate^22^,^23^,^24^,^25^,^29^, giving rise to a limited carrier mobility^32^. On one hand the lower mobility reduces the chance that carriers reach contacts in the applied bias field before recombination, resulting in a limited contribution from the photo-electric effect (short blue arrow in Fig. 5a). On the other hand the SPP scattering and charged impurity scattering^33^,^34^ effectively cools the carriers, while they diffuse and drift in the field towards contacts, giving rise to a large spatial gradient ΔT_e_ in carrier temperatures. This ΔT_e_ is the drive for the Seebeck effect and thus a strong photo-thermoelectric contribution (long red arrow in Fig. 5a).

In contrast, for suspended graphene the SPP interaction is absent and charged impurity scattering is strongly reduced between source and drain contacts, giving rise to much higher carrier mobility^35^,^36^,^37^. On one hand this improves the chance that photoexcited carriers separate and drift towards contacts in the applied electric field, resulting in a larger contribution of the photo-electric effect (long blue arrow in Fig. 5b). On the other hand the flexural phonons (ZA), which are suppressed by the substrate in supported graphene, are present in suspended graphene and therefore dominate the heat conduction^38^,^39^,^40^. The larger heat conduction in suspended graphene reduces the temperature gradient between the excitation spot and the metal contacts, corresponding to an effective slow-down of carrier cooling, thereby reducing the photo-thermoelectric contribution (short red arrow in Fig. 5b).

Quantitatively, the photoelectric contribution depends linearly on photogenerated carrier density which is directly proportional to the rate of absorbed photons and thus the incident excitation power. Therefore, the photoelectric contribution I_PE_ should be linearly proportional to the incident power P_opt_, i.e. α = 1. The observed power exponent of α = 0.9 ± 0.05 in the experiment of our fully suspended graphene device is in good agreement with a dominant contribution from the photo-electric effect. We attribute the attenuation of the value of α from unity to 0.9 to the photo-thermoelectric (α = 0.66) effect still partly contributing to the photocurrent in this device. In addition, Fig. 4 shows that the photocurrent saturation occurs at six-fold lower laser power values in the fully suspended graphene device than those in the supported device (horizontal arrow marked “A”). Saturation sets in when all energy states of the hot electrons in the conduction band around the pump energy are populated by the photoexcited carriers. This effect is particularly pronounced if a bottleneck exists for carrier cooling/relaxation, as is expected for suspended graphene, leading to a reduced contribution of the photo-thermoelectric effect, and thus implying larger contributions of the photoelectric effect. Both, the six-fold earlier onset of the saturation behavior at about 80 μW pump power and the slope of the L-I curve close to unity strongly indicate that the photocurrent generation in our fully suspended graphene device is dominated by the photoelectric effect.

In contrast to our findings, a recent study of partially supported graphene shows that such a sample design yields a dominant photo-thermoelectric effect^29^. Partially supported means that next to the suspended graphene area, a region several micrometers long of substrate-supported graphene exists, which can give rise to efficient carrier cooling via substrate interactions and thus a large spatial ΔT_e_, driving the Seebeck effect before reaching contacts. It is thus evident that novel graphene photodetectors can be engineered in which either the photo-thermoelectric or the photoelectric effect dominates the photoresponse. Since the time constant of the photoelectric effect is, at 4 ps, over an order of magnitude faster than the time constant of the photo-thermoelectric effect, at 130 ps^26^, another advantage of our fully suspended device design beyond the demonstrated four-fold enhanced photoresponsivity is a potentially higher cut-off frequency in the dynamic response near THz frequencies.

In conclusion, we have demonstrated four-fold enhancement of the photoresponsitivity of fully suspended CVD-grown graphene photodetector devices over substrate-supported graphene. In addition, we have shown that the light-current input/output curves give valuable information on the underlying photophysical process responsible for the generated photocurrent. Finally, we have found that fully suspended CVD-grown graphene devices are dominated by the photoelectric effect, which is promising towards CVD-grown graphene photodetectors approaching THz cut-off frequencies.

## Methods

### Device fabrication

Large area graphene was grown on high purity copper foil using a low pressure CVD furnace at 1000°C in a hydrogen, argon and methane environment. Raman spectrum of the graphene transferred onto 300 nm SiO_2_ substrate confirms good quality monolayer graphene film (For more details see supporting online information.). The graphene was then etched into ribbons using reactive ion etching with O_2_. The patterned graphene microribbons were then contacted with e-beam evaporated 125 nm thick Ti/Au contact pads, defined using e-beam lithography of PMMA resist and lifted off by N-Methyl-2-pyrrolidone (NMP). The devices were then wire bonded using 25 μm thick Au wires on a chip carrier for electrical measurements. For suspending graphene, we etched the 300 nm thick underlying layer of SiO_2_ using (6:1) buffered oxide etchant (BOE) followed by critical point drying (CPD) to avoid damage to the suspended graphene ribbon.

### Electrical and photocurrent measurements

All the measurements were performed at room temperature in a chamber with vacuum level of 10^−6^ mbar. The photoresponse of the graphene ribbons was measured using a lock-in amplifier by positioning a 532 nm wavelength laser spot onto the ribbon. A spot of approximately 1 μm diameter (FWHM) was focused using a 50 × objective with working distance of 13 mm. The position of the laser spot was manipulated by using piezoelectric motor controlled scanning mirrors with nanometer precision in X and Y directions. The incident laser power, which was measured to be 20 mW at maximum, was controlled by using neutral density filters to sweep between 1 nW and 2.8 mW. The beam was modulated with a chopper wheel at 2 kHz. All current and voltage values were supplied by two Keithly 2400 source meters, while output was read from the lock-in amplifier. A moderate source-drain bias of 0.1 V was used for photocurrent measurements. Back-gate sweeps were performed with 1 mV with voltage ranges selected to center the Dirac point.

## Author Contributions

V.P., S.S. and E.H.Y. conceived and designed the experiments. V.P fabricated the suspended CVD-graphene microribbons. V.P. carried out the electro-optical experiments and analyzed the data. V.P. and A.C. carried out the Raman experiments. V.P., S.S. and E.H.Y. co-wrote the manuscript. E.H.Y. supervised the device fabrication and S.S. supervised the electro-optical measurements. All authors contributed to scientific discussions.

## Supplementary Material

Supplementary Information SI
