# Supplementary material for: Improved photoresponse with enhanced photoelectric contribution in fully suspended graphene photodetectors
Source: Sci Rep. 2013 Sep 27;3:2791. doi: 10.1038/srep02791 (PMC3784941; doi:10.1038/srep02791)
Supplement: Supplementary Information — SI [file srep02791-s1.pdf]

Supplementary information for "Improved photoresponse with enhanced photoelectric contribution in fully suspended graphene photodetector"

Vikram Patil[†], *Aaron Capone* [§], *Stefan Strauf* [§], *and Eui-Hyeok Yang* [†*]

† Department of Mechanical Engineering, Stevens Institute of Technology

Castle Point on the Hudson, Hoboken, NJ 07030, USA

§Department of Physics & Engineering Physics, Stevens Institute of Technology

Castle Point on the Hudson, Hoboken, NJ 07030, USA

SUPPLEMENTARY FIGURES/METHODS

Graphene growth using LPCVD

The graphene growth process was performed on a 25 µm thick, high purity Cu foil (Alfa Aesar) in an 80 mm diameter tube furnace of a low-pressure chemical vapor deposition (LPCVD) system. The Cu foil was heated to 1000 °C in a hydrogen and argon environment (H_2:Ar, 240:1000sccm). After stabilization period of 30 minutes at 1000 °C, methane (CH_4, 36 sccm) was introduced in the furnace for 5 minutes. Then the furnace was rapidly cooled down to room temperature in the hydrogen and argon environment. After the cool down of the Cu foil, the furnace was brought to atmospheric pressure. The graphene-coated Cu foil was further processed to etch away the Cu film and transferred onto a SiO_2 substrate.

Transfer of LPCVD-grown graphene and optical transmission measurement

To transfer the graphene layer onto a substrate, the copper layer was etched away in a copper etchant bath using a commercially available etchant solution (Transene 49-1) as shown in Fig. S1a. After copper was removed, the graphene was transferred into a distilled (DI) water bath (Fig. S1b) to remove any trace of the etchant. After three DI water bath transfers, one part of the clean graphene layer was transferred onto a Si substrate with 300 nm SiO_2 for photodetector device fabrication and another part onto a clean glass slide (Fig. S1c) for optical transmission measurements. To confirm the quality of LPCVD-grown graphene over a large-area after the transfer process, optical transmission measurements were carried out (Fig, S1d). For the measurements, white light was focused with a microscope objective onto the graphene/glass sample and the transmitted light was collected with another microscope objective and sent into a spectrometer. The spectrum was normalized to the transmission of the bare glass substrate. The normalized data was smoothed by adjacent averaging using 500 points. An average of ~97% universal transmission through various locations on the graphene sample shows good, consistent quality of the monolayer CVD graphene.

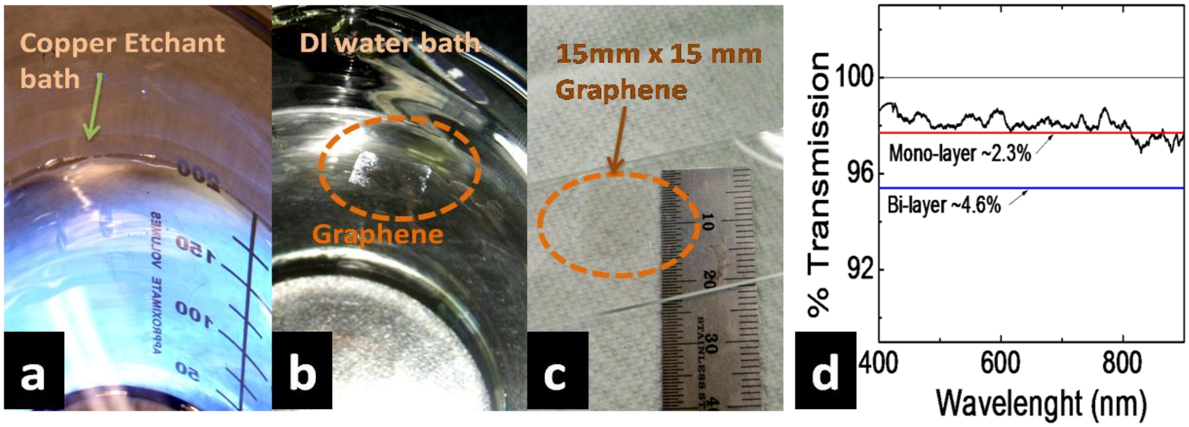

Supplementary Figure S1: Optical transmission measurements through graphene on a glass substrate. **a,b,c**. CVD-grown graphene transfer onto a glass slide after processing through a copper etchant and two DI water baths. **d.** Transmission measurements on the glass slide.

Raman spectrum of graphene

Graphene was transferred onto a 300 nm SiO_2 substrate to evaluate its quality using Raman spectroscopy. Raman measurements were taken with a 523 nm laser focused to a spot diameter of ~2 μm (FWHM) onto the graphene layer using a 100x objective. The signal was accumulated for 5 seconds and summed over five consecutive spectra. Figure S2 shows the characteristic graphene-Raman peaks G and G' at 1570 cm^{-1} and 2650 cm^{-1}, respectively. A G' peak width (FWHM) of 34 cm^{-1} and $I(G')/I(G) > 1$ confirms monolayer graphene[41,42]. The low intensity ratio $I(D)/I(G)$ also indicates a fairly low defect density in the graphene layer.

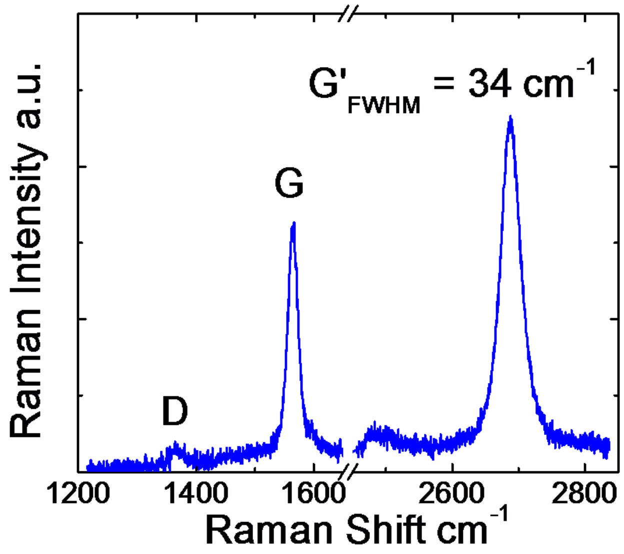

Supplementary Figure S2: Raman signal of the large area graphene transferred onto a 300nm SiO_2 substrate showing typical Raman peaks of graphene.

Fabrication of the graphene devices

Graphene ribbons were fabricated to be 2 μm wide and 5 μm long between the metal contacts. To etch the graphene ribbon, target graphene areas were protected using a nickel layer which was patterned and deposited using a JEOL6300 e-beam lithography system and a Kurt-Lesker e-beam evaporator at Brookhaven National Laboratory. After the graphene etching using the Trion Tech RIE system, the nickel layer was etched away using standard nickel etchant. We chose to use a nickel mask for graphene etching since it does not contaminate graphene ribbons unlike other resist materials such as PMMA as shown in recent study by Kumar *et al*[43]. After removal of the nickel layer, the etched graphene sample was rinsed with DI water thoroughly to remove nickel etchant and carefully dried with nitrogen. Then the sample was further vacuum annealed at 250 °C for 2 hours to remove any residue of water molecules from the surface of the etched graphene following Cheng *et al*[44]. The resulting surface of the etched graphene was thus clean enough to allow reliable fabrication of well conducting metal contact pads.

Fabrication of the suspended devices

Figure S3a shows the device geometry with each graphene ribbon contacted using individual pairs of source and drain electrodes. Each electrode was then wire-bonded to individual pins and a bias was applied separately for each source-drain pair. This avoided cross-talk between neighboring devices, especially during the backgated photocurrent measurements. Figure S3-b shows the profile of an under-etched metal electrode after etching the SiO_2 with buffered oxide etchant (BOE) for 4 minutes followed by a critical point drying (CPD) process to avoid stiction. The well-defined under-etching ensures that the suspended graphene ribbon is well isolated from the p-doped silicon substrate by an approximately 300 nm air-gap. The CPD process was carried

out using high purity ethanol as a transfer chemical and liquid CO_2 as a sample drying chemical. During the CPD process, the chamber was pressurized and heated above the critical point of CO_2 and then slowly de-pressurized such that the drying of the sample occurred without any phase-change of CO_2 avoiding any damage to the suspended graphene microribbons. The rate of de-pressurization of the chamber was carefully controlled to avoid condensation of any trapped impurities on the surface of the device.

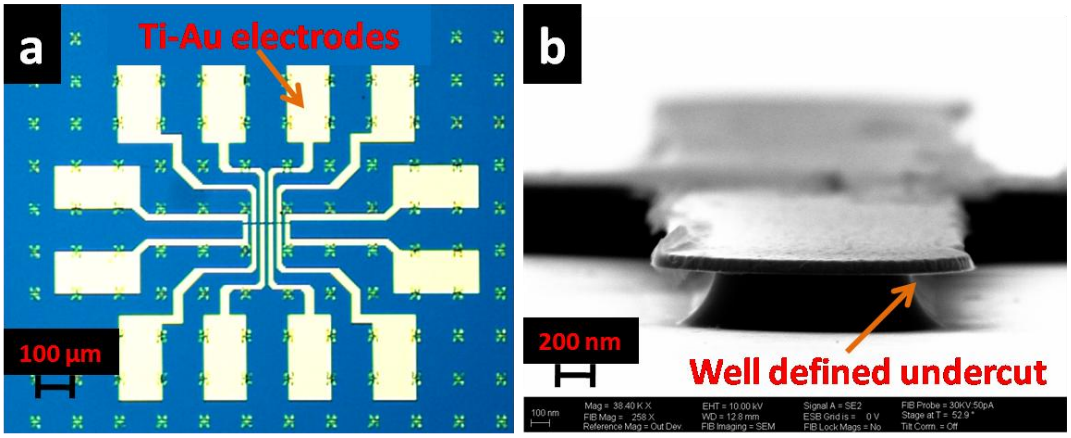

Supplementary Figure S3: a. Graphene photodetector device as fabricated for this work, **b.** Well-defined profile of under-etched metal electrode.

Current and laser annealing of graphene ribbons

The as-fabricated graphene devices in this study typically show heavy p-doping of the graphene layer. Incident laser excitation with current flowing through the graphene ribbon has been shown to remove the p-dopants from the graphene layer, shifting the Fermi-level towards the intrinsic Dirac point[45]. Therefore, laser-current annealing was done on as-fabricated graphene devices before the photocurrent measurements to avoid unwanted shift in the photocurrent values during the measurements due to the shift in the Dirac point. Figure S3 shows a significant shift in the

Dirac point after laser-current annealing. This indicates effective removal of dopants from the surface of the graphene ribbon and leading to enhanced mobility and reduced hysteresis[43]. A stable Dirac point at around +10 V was obtained after 12 hours of annealing.

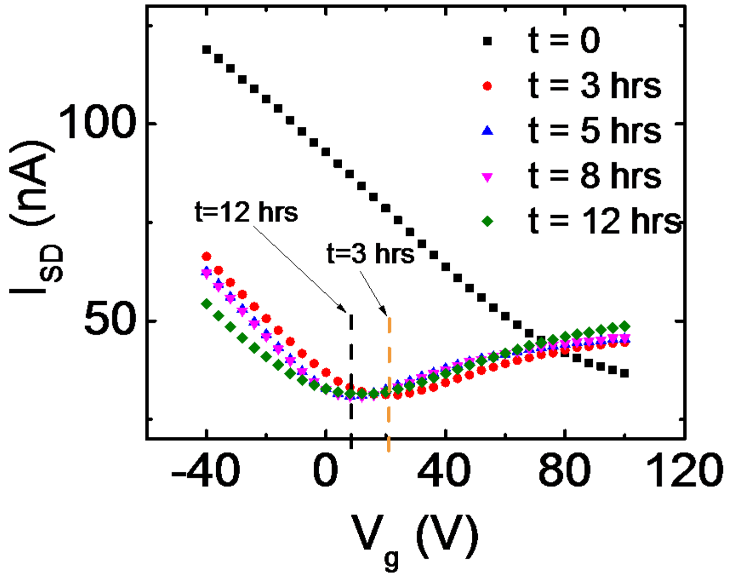

Supplementary Figure S5: Laser-current annealing with laser power = 1 mW on the graphene ribbon and source drain current of 2 μA. A current versus backgate voltage measurement was performed at different time intervals to record the shift of the Dirac point. A stable Dirac point around +10 V was obtained after 12 hours of annealing.

Raman spectrum of the suspended graphene

Figure S5 shows the 2D Raman scan on the suspended graphene held in air. The two-dimensional Raman scans of the G peak clearly show uniform quality of the suspended graphene ribbon areas, i.e. no indication for pronounced spatial inhomogeneities caused by residual unintentional doping.

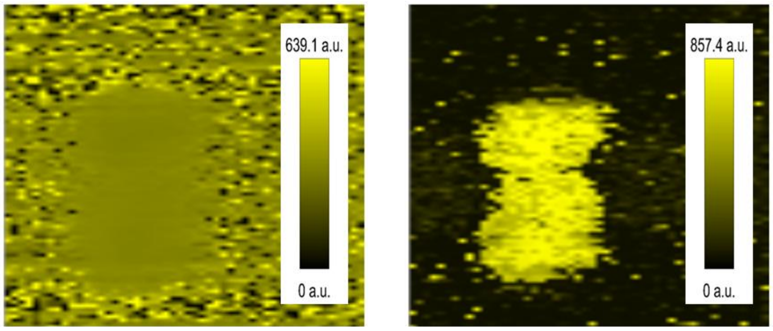

(a) Raman G peak position relative to the background @ ~1570 cm^{-1}

(b) Raman G peak width relative to the background @ ~1570 cm^{-1}

Supplementary Figure S6: Raman scan on a suspended graphene ribbon held in air (a) G peak position map at 1570 cm^{-1}, (b) G peak width map at 1570 cm^{-1}

SUPPLEMENTARY DISCUSSION

Estimation of light-current curve power exponents:

a) For the photoelectric effect:

From a simple model[46] of a photodetection layer, a steady state photocarrier generation rate per unit volume (G_e) is given as:

$$G_e = \eta \left(\frac{P_{opt}}{h\nu} \right) \frac{1}{A_{Laser} D} \qquad \text{SE (1)}$$

where, η is the quantum efficiency of the photodetector layer, P_{opt} is the incident power, A_{Laser} is the area under the laser excitation spot, D is the layer thickness and hv is the energy of the incident photon. For steady state, the photocarrier density is given as:

$$n = G_e \, \tau \qquad \text{SE (2)}$$

where τ is an average lifetime of the photo-generated carriers. Combining the supplementary equations SE (1) and SE (2), the steady state photocarrier density can be evaluated as:

$$n = \eta \left(\frac{P_{opt}}{h\nu} \right) \frac{1}{A_{Laser} D} \tau. \qquad \text{SE (3)}$$

The photocurrent generated in the photodetection layer can then be expressed using the following equation:

$$I_{PE} = \sigma_p \, \xi \, WD \qquad \text{SE (4)}$$

where σ_p is conductivity of the photodetector layer due to the photo-generated carriers, ξ is an applied electric field across the layer and W is width of the layer. Thus, the photocurrent measured at the source and drain contacts with a carrier separation efficiency β is:

$$I_{PE} = \beta \left(\mu_n + \mu_p\right) nq \;\xi\, WD \qquad \text{SE (5)}$$

where μ_n and μ_h are the electron and the hole mobility respectively and q is the electron charge. Substituting n in the supplementary equation SE (3) into equation SE (5), we obtain

$$I_{PE} = q\,\eta\,\beta \left(\frac{P_{opt}}{h\nu}\right) \frac{(\mu_n + \mu_p)W}{A_{Laser}}\,\tau\,\xi. \qquad \text{SE (6)}$$

As the electric field across the length (L) of the layer is related to the applied bias voltage (V) via $\xi = V/L$, the supplementary equation SE(6) can be re-written as:

$$I_{PE} = q\,\eta\,\beta \left(\frac{P_{opt}}{h\nu}\right) \frac{(\mu_n + \mu_p)W}{L * A_{Laser}}\,\tau\,V.$$

With this model, the relationship between the photoelectric current generated in a photodetector layer and the incident power can be expressed as:

$$\boldsymbol{I_{PE} \propto P_{opt}}$$

b) For the photo-thermoelectric effect:

The thermoelectric current originates from carrier diffusion when a thermal gradient is generated between the photoexcited electrons at the excitation spot and the graphene-metal interface. The thermoelectric contribution can be mathematically expressed as:

$$I_{TE} \propto S(T) * \Delta T_e \qquad \text{SE (7)}$$

where *S(T)* is the thermoelectric power which can be obtained using the Mott formula[47]. *S(T)* is directly proportional to the hot electron temperature term T_e such that $S \propto T_e$. The term ΔT_e is proportional to the hot electron temperature difference between the excitation area and near the contacts. The portion of the incident energy received by the hot electrons is proportional to the incident power which results in $C_e T_e \propto P_{opt}$. The term C_e is the heat capacity of the electrons which is temperature dependent[48] such that $C_e \propto T_e{}^2$. Therefore, the electron temperature is related to the incident power as: $T_e{}^3 \propto P_{opt}$ or $T_e \propto P_{opt}{}^{\frac{1}{3}}$. Thus from the supplementary equation SE (7), the relationship between the thermoelectric current and the incident power P_{opt} can be expressed as:

$$I_{TE} \propto (P_{opt})^{2/3}.$$

SUPPLEMENTARY REFERENCES

41 Malard, L. M., Pimenta, M. A., Dresselhaus, G. & Dresselhaus, M. S. Raman spectroscopy in graphene. *Physics Reports* **473**, 51-87 (2009).

42 Bhaviripudi, S., Jia, X., Dresselhaus, M. S. & Kong, J. Role of Kinetic Factors in Chemical Vapor Deposition Synthesis of Uniform Large Area Graphene Using Copper Catalyst. *Nano Letters* **10**, 4128-4133 (2010).

43 Kumar, S. *et al.* Reliable processing of graphene using metal. *Nanoscale Research Letters* **6** (2011).

44 Cheng, Z. et al. Toward Intrinsic Graphene Surfaces: A Systematic Study on Thermal Annealing and Wet-Chemical Treatment of SiO2-Supported Graphene Devices. *Nano Letters* **11**, 767-771 (2011).

45 Freitag, M., Low, T., Xia, F. & Avouris, P. Photoconductivity of biased graphene. *Nat Photon***7**, 53-59 (2013).

46 Sze S.M. & Ng K.K. *Physics of Semiconductor Devices, 3rd edn,* (Wiley-Interscience, 2006).

47 Jonson, M. & Mahan, G. D. Mott's formula for the thermopower and the Wiedemann-Franz law. *Physical Review B* **21**, 4223-4229 (1980).

48 Sun, D. *et al.* Ultrafast hot-carrier-dominated photocurrent in graphene. *Nat Nano* **7**, 114-118 (2012).
